# A highly potent and broadly accessible bispecific nanobody for the treatment of ebola virus infections

**DOI:** 10.1371/journal.ppat.1013878

**Published:** 2026-01-27

**Authors:** Fan Bu, Gang Ye, Kimberly Morsheimer, Hailey Turner-Hubbard, Brett Eaton, Manu Anantpadma, Khaggeswar Bheemanapally, Chalet Tan, Robert Davey, Fang Li

**Affiliations:** 1 Department of Pharmacology, University of Minnesota Medical School, Minneapolis, Minnesota, United States of America; 2 Center for Emerging Viruses, University of Minnesota, Minneapolis, Minnesota, United States of America; 3 National Emerging Infectious Diseases Laboratories, Boston University, Boston, Massachusetts, United States of America; 4 Department of Virology, Immunology, and Microbiology, Boston University School of Medicine, Boston, Massachusetts, United States of America; 5 Integrated Research Facility at Fort Detrick, Division of Clinical Research, National Institute of Allergy and Infectious Diseases, National Institutes of Health, Frederick, Maryland, United States of America; 6 Department of Pharmaceutical Sciences, University of Tennessee Health Science Center, Memphis, Tennessee, United States of America; University of Iowa, UNITED STATES OF AMERICA

## Abstract

Ebola virus (EBOV) causes recurring outbreaks, with a case fatality rate of about 40%. Currently approved vaccine and antibody therapies face major limitations, including only modest reductions in mortality and restricted accessibility due to their reliance on injection-based delivery and cold-chain transport and storage. To address these challenges, we developed a bispecific nanobody, Nanosota-EB1/EB2-Fc, composed of two nanobodies (camelid-derived single-domain antibodies, Nanosota-EB1 and Nanosota-EB2) that target distinct epitopes on the EBOV glycoprotein (GP) and are fused to a human Fc domain. Through cooperative contributions from both nanobodies, this bispecific nanobody strongly inhibits GP function and effectively overcomes the virus’s decoy mechanism. A single dose provided strong protection in EBOV-infected mice, including when administered at late stages of infection. It was also effective when administered intranasally, offering a needle-free delivery option. Furthermore, its high in vitro stability indicates that it can be deployed without refrigeration. Taken together, this novel bispecific nanobody represents a promising next-generation therapeutic for EBOV, combining high potency with broad accessibility.

## Author summary

Ebola virus (EBOV) remains a significant global health threat, with current treatments providing only modest survival benefits and limited accessibility due to their dependence on injection and cold-chain transport and storage. Here we developed a novel bispecific nanobody, Nanosota-EB1/EB2-Fc, which combines two nanobodies (single-domain antibodies) targeting distinct epitopes on the EBOV glycoprotein. Through intramolecular cooperativity, Nanosota-EB1/EB2-Fc inhibits glycoprotein function and evades the virus’s decoy mechanisms. A single dose showed strong therapeutic efficacy in EBOV-infected mice, including when administered at late stages of infection. Nanosota-EB1/EB2-Fc is also suitable for intranasal administration and exhibits high in vitro thermostability, enabling needle-free delivery and refrigeration-independent deployment. Together, these features position Nanosota-EB1/EB2-Fc as a highly potent and broadly accessible therapeutic candidate for future EBOV outbreaks.

## Introduction

Ebola virus (EBOV) is one of the deadliest known pathogens, with a case fatality rate of ~40%. It causes severe hemorrhagic fever, a major factor contributing to its high mortality. To date, EBOV has infected more than 33,000 people, including 28,652 cases during the unprecedented West African outbreak from 2014 to 2016 [1,2]. Smaller outbreaks of EBOV and related filoviruses continue to occur regularly [3], and a recent outbreak in the Democratic Republic of the Congo underscores the persistent risk [4]. It is believed that EBOV has natural reservoirs, although the virus has yet to be definitively identified in the wild [5]. Moreover, EBOV can persist in the human body for years in a dormant state before reactivating and causing new infections [6]. These factors together contribute to the virus’s recurrent emergence. Given its high lethality and long-term persistence in human hosts, EBOV remains a serious threat to global health and national security.

Current interventions for EBOV infections have major limitations. The only FDA-approved vaccine reduces the fatality rate to approximately 25% - a level that remains unacceptably high [7]. Additionally, the two FDA-approved antibody therapies lower mortality to around 35%, including in some late-stage cases - a cause for concern [8,9]. Both the vaccine and antibody therapies require injection-based administration and cold-chain transport and storage, severely limiting their accessibility - especially in remote, resource-limited, and warm-climate regions where EBOV outbreaks typically occur [10]. As a result of these logistical challenges, only 41% of eligible patients received antibody therapies during recent outbreaks in the Democratic Republic of Congo and Guinea [11]. These limitations underscore the urgent need for highly potent and broadly accessible therapies to effectively combat EBOV infections.

The EBOV glycoprotein (GP) is the primary target for neutralizing antibodies, as it mediates viral entry into human cells [12]. On the viral surface, GP exists in a metastable “pre-fusion” trimeric form, composed of three copies each of the receptor-binding subunit GP1 and the membrane-fusion subunit GP2 [13]. GP1 contains the receptor-binding site (RBS), which is shielded by a glycan cap and a mucin-like domain (MLD) [12,14]. GP2 contains a fusion peptide and two heptad repeat regions (HR1 and HR2) [15]. To initiate infection, GP1 first binds to host cell surface factors, leading to EBOV internalization via endocytosis. Within endosomes, the glycan cap and MLD are removed by proteolytic cleavage, exposing the RBS [16]. The exposed RBS then binds to its cellular receptor, Niemann-Pick C1 (NPC1), on the endosomal membrane [17,18]. This interaction triggers a dramatic conformational change in GP2, transitioning it to its most stable “post-fusion” form and enabling fusion of the viral and endosomal membranes [19,20]. Antibodies targeting EBOV GP can, in principle, disrupt any of these entry steps [21–23]. In addition, antibodies that recognize exposed GP epitopes, particularly those distal to the viral membrane, can recruit Fc-mediated effector functions such as antibody-dependent cellular cytotoxicity (ADCC) by bridging GP and Fcγ receptors (FcγRs) on immune cells, thereby activating antiviral immune responses [23–26]. Soluble GP (sGP), a decoy protein encoded by EBOV and secreted by infected cells, presents an additional complication for antibody-based therapies. sGP comprises most of GP1 but lacks GP2, and can bind and sequester GP1-targeting antibodies, diverting them from the functional GP on the viral surface [27,28]. Antibodies that interfere with GP function while limiting diversion by sGP are therefore likely to be potent neutralizers of EBOV entry. Additionally, antibodies that recognize exposed GP epitopes can contribute to protection by engaging Fc-dependent antiviral effector mechanisms, such as ADCC.

Nanobodies are single-domain antibodies derived from the heavy-chain-only antibodies of camelid animals [29,30]. Their unique structure offers several therapeutic advantages for antiviral applications. Nanobodies can achieve high antiviral potency, owing to their excellent epitope accessibility and tissue penetration [31,32]. They are also cost-effective to produce, transport, and store, and have the potential for intranasal administration [33,34]. In addition, they exhibit minimal host toxicity due to their high specificity for viral targets, and minimal immunogenicity in humans due to their strong similarity to human germline antibodies [31,32]. In 2019, the FDA approved the first nanobody-based therapeutic for the treatment of a blood clotting disorder [35]. Since the onset of COVID-19, we have developed nine nanobody inhibitors, collectively known as the Nanosota series, that target the SARS-CoV-2 spike protein [34,36–39]. More recently, we developed two nanobodies, Nanosota-EB1 and Nanosota-EB2, which are the first nanobodies ever generated against EBOV [40]. These nanobodies bind distinct epitopes on EBOV GP and inhibit its function through different mechanisms: Nanosota-EB1 recognizes the glycan cap of GP1, slowing proteolytic cleavage and delaying exposure of the RBS, and its highly exposed epitope suggests substantial ADCC potential; in contrast, Nanosota-EB2 targets key membrane-fusion elements in GP2, blocking the conformational transition required for membrane fusion, but its less exposed epitope suggests limited ADCC potential [40]. Together, these nanobodies establish a foundation for nanobody-based therapies to treat EBOV infection.

Despite their promise as next-generation anti-EBOV therapeutics, several key questions remain regarding Nanosota-EB1 and Nanosota-EB2: Can they cooperatively block GP function? Can their antiviral potency be maximized through a bispecific design? And can their unique structural properties broaden their accessibility? These questions are addressed in the present study.

## Results

### Design and in vitro characterization of anti-EBOV bispecific nanobody

**Fig 1:**
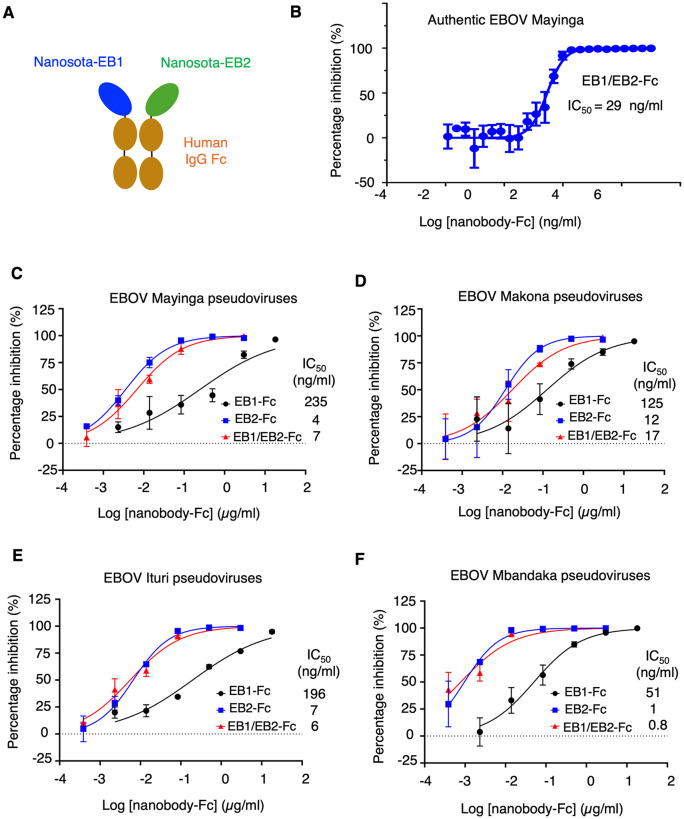
Construction of the bispecific nanobody and characterization of its in vitro anti-EBOV potency. **(A)** Construction of the bispecific nanobody Nanosota-EB1/EB2-Fc by fusing two individual nanobodies, Nanosota-EB1 and Nanosota-EB2, to a human Fc domain. **(B)** Efficacy of Nanosota-EB1/EB2-Fc in neutralizing live EBOV infection. Authentic EBOV (Mayinga strain) was used to infect Huh7 cells in the presence of Nanosota-EB1/EB2-Fc at different concentrations. Neutralization efficacy was expressed as the concentration required to reduce EBOV infection by 50% (IC_50_). Error bars represent SEM (n = 3). **(C)-(F)** Efficacy of Nanosota-EB1/EB2-Fc in neutralizing EBOV pseudoviruses. Retroviruses pseudotyped with full-length EBOV GP from four different EBOV strains were used to infect Huh7 cells in the presence of Nanosota-EB1/EB2-Fc at different concentrations. Neutralization efficacy was expressed as the concentration required to reduce pseudovirus entry by 50% (IC_50_). Error bars represent SEM (n = 3).

Of the two anti-EBOV nanobodies we recently discovered, Fc-domain-fused Nanosota-EB2 (EB2-Fc) potently neutralizes both EBOV pseudoviruses (retroviral particles pseudotyped with EBOV GP) and authentic EBOV, whereas Fc-domain-fused Nanosota-EB1 (EB1-Fc) shows moderate neutralizing potency against EBOV pseudoviruses but only weak activity against authentic virus [40]. This markedly reduced activity of EB1-Fc against authentic EBOV likely reflects diversion by sGP, which binds EB1 but not EB2 [40]. To evaluate whether EB1 and EB2 can cooperatively inhibit EBOV, we designed a bispecific nanobody, Nanosota-EB1/EB2-Fc (EB1/EB2-Fc), in which both nanobodies are fused to a human IgG Fc domain (Fig 1A). To promote formation of the bispecific nanobody, we introduced two Fc-region mutations: T366Y into the EB1-Fc construct and Y407T into the EB2-Fc construct [41]. This “knobs-into-holes” strategy facilitates Fc heterodimerization because pairing of the knob (Y366) with the hole (T407) is far more stable than knob/knob or hole/hole pairings, leading to highly efficient heterodimer formation (>90%) as reported previously [41–47]. EB1/EB2-Fc was expressed in Expi293F mammalian cells at yields exceeding 50 mg/L of culture medium, comparable to those obtained for EB1-Fc and EB2-Fc [40]. The purified EB1/EB2-Fc binds both sGP (via EB1) and GP lacking the glycan cap (via EB2) and also displays remarkable in vitro stability (see below for both properties), confirming successful heterodimer assembly and aligning with previous extensive analyses showing that the knob/hole heterodimer is far more stable than either knob/knob or hole/hole homodimers.

We next assessed the potency of EB1/EB2-Fc in neutralizing EBOV entry in vitro. Against authentic EBOV (Mayinga strain), EB1/EB2-Fc exhibited an IC₅₀ of 29 ng/ml (Fig 1B), comparable to the neutralization potency of EB2-Fc (IC₅₀ = 47 ng/ml) and markedly greater than that of EB1-Fc (IC₅₀ = 47 μg/ml) reported previously [40]. We then evaluated EB1/EB2-Fc against EBOV pseudoviruses, with EB1-Fc and EB2-Fc tested in parallel. In addition to the prototypic Mayinga strain, three other EBOV strains (Makona, Ituri, and Mbandaka) were examined. EB1/EB2-Fc demonstrated IC₅₀ values ranging from 0.8 to 17 ng/ml against these pseudoviruses, again comparable to EB2-Fc (IC₅₀ = 1–12 ng/ml) and substantially more potent than EB1-Fc (IC₅₀ = 51–235 ng/ml) (Fig 1C-1F). Theoretically, as a hybrid, EB1/EB2-Fc (containing one copy each of EB1 and EB2) would be expected to show intermediate potency between EB1-Fc (containing two copies of EB1) and EB2-Fc (containing two copies of EB2). Instead, EB1/EB2-Fc displays anti-EBOV activity similar to EB2-Fc and far exceeding EB1-Fc, indicating cooperative contributions of EB1 and EB2 within the EB1/EB2-Fc molecule. Moreover, EB1/EB2-Fc exhibits broad-spectrum anti-EBOV activity, potently neutralizing all four major EBOV strains tested.

**Fig 2:**
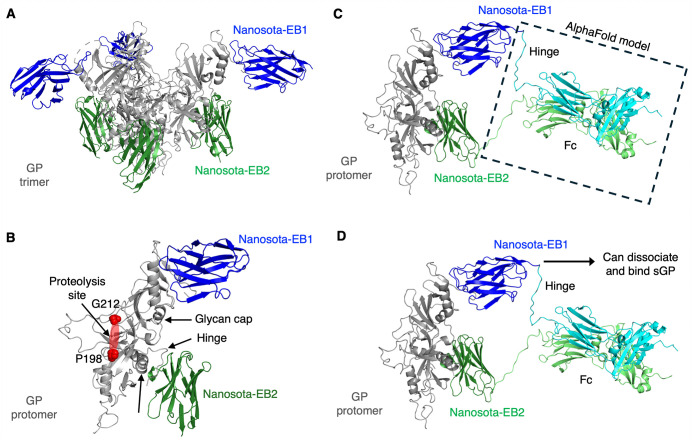
Structural basis for cooperative binding of EB1 and EB2 to EBOV GP and a model for dual engagement by EB1/EB2-Fc. **(A)** Cryo-EM structure of the ternary complex consisting of EBOV GP ectodomain, EB1, and EB2. The trimeric GP ectodomain is shown in gray; the three EB1 molecules are shown in blue; and the three EB2 molecules are shown in green. **(B)** Close-up view of the cooperative binding of EB1 and EB2 to EBOV GP. EB2 binding indirectly stabilizes the glycan cap via a hinge region. The proteolysis loop (residues 198-212), which is disordered in the structure, is indicated by a red oval. **(C)** Model of Fc-linked bispecific nanobody engagement on a single GP protomer. The cryo-EM protomer bound to EB1 and EB2 is shown alongside an AlphaFold model of the human IgG1 Fc region (AlphaFold database identifier: AF-P0DOX5-F1; dashed box), illustrating how the Fc hinge can enable simultaneous engagement of the EB1 and EB2 epitopes on the same protomer. **(D)** Schematic model illustrating resistance of EB1/EB2-Fc to sGP-mediated diversion. EB1/EB2-Fc can remain anchored to GP via EB2 while EB1 engages sGP, limiting diversion by the sGP decoy while preserving GP engagement.

To understand the cooperative inhibition of EBOV entry by the bispecific nanobody, we determined the cryo-EM structure of EBOV GP in complex with both EB1 and EB2. Our previous cryo-EM studies of GP bound to individual nanobodies revealed that each trimeric GP was bound by either two EB1 molecules or three EB2 molecules [40]. The limited occupancy of EB1 (only two per trimer) was likely due to the flexibility of the EB1-bound glycan cap, rather than weak binding affinity, since EB1 binds GP with a Kd of 2.77 nM, indicating strong interaction. Surprisingly, in the GP/EB1/EB2 ternary complex structure, each trimeric GP was bound by three EB1 molecules and three EB2 molecules (Fig 2A and S1 Fig and S1 Table). This finding suggests that the simultaneous binding of EB1 and EB2 stabilizes the glycan cap, allowing full EB1 occupancy and demonstrating cooperative binding of both nanobodies to GP. Further structural analysis indicated that EB2 binding to GP2 indirectly stabilizes the glycan cap via a hinge region (Fig 2B). Because glycan cap stabilization has been associated with reduced proteolytic cleavage [14,40], these data point to a mechanism by which EB1/EB2-Fc more effectively suppresses glycan cap removal. AlphaFold modeling further supports this model, indicating that the Fc-linked bispecific construct can simultaneously engage the EB1 and EB2 epitopes on the same GP protomer (Fig 2C), thereby stabilizing the glycan cap and impeding its proteolysis. The model also suggests that EB1/EB2-Fc may bind GP via EB2 while engaging soluble GP (sGP) via EB1 (Fig 2D), potentially limiting diversion by the sGP decoy. Together, the GP/EB1/EB2 ternary structure provides a framework for understanding how the bispecific nanobody achieves cooperative inhibition of EBOV infection.

**Fig 3:**
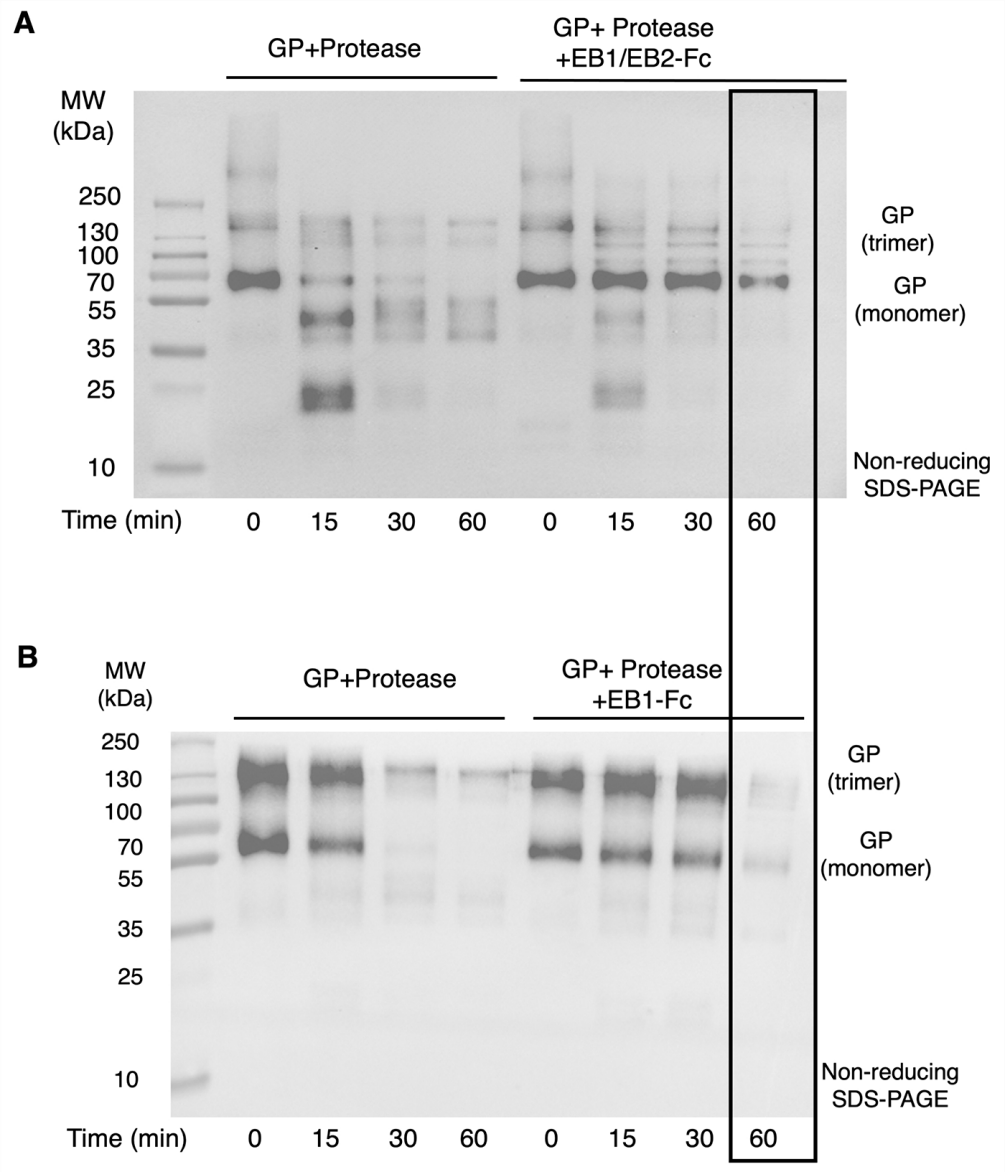
Cooperative activity of the bispecific nanobody in impeding proteolysis of the EBOV GP glycan cap. **(A)** Glycan cap cleavage assay assessing the effect of EB1/EB2-Fc on glycan cap proteolysis, using Western blot detection of the His tag on GP ectodomain under non-reducing conditions. EB1/EB2-Fc inhibited GP ectodomain proteolysis after 60 minutes (boxed). The experiment was repeated three times with consistent results. **(B)** Glycan cap cleavage assay assessing the effect of EB2-Fc on glycan cap proteolysis, using the same assay conditions as in panel **(A)**. EB1-Fc inhibited GP ectodomain proteolysis for up to 30 minutes. The result in panel (B) was reported in our previous publication [40] and is shown here for comparison with panel **(A)**.

To test whether the bispecific nanobody enhances inhibition of glycan cap proteolysis, we conducted a time-course glycan cap proteolysis assay with EB1/EB2-Fc (Fig 3A). Our previous work showed that EB1-Fc effectively inhibited glycan cap proteolysis for up to 30 minutes (Fig 3B) [40]. In contrast, the current study found that EB1/EB2-Fc maintained efficient inhibition even after 60 minutes (Fig 3A). These results demonstrate that the bispecific nanobody is more effective than EB1-Fc at inhibiting glycan cap proteolysis, owing to the cooperative action of EB1 and EB2.

**Fig 4:**
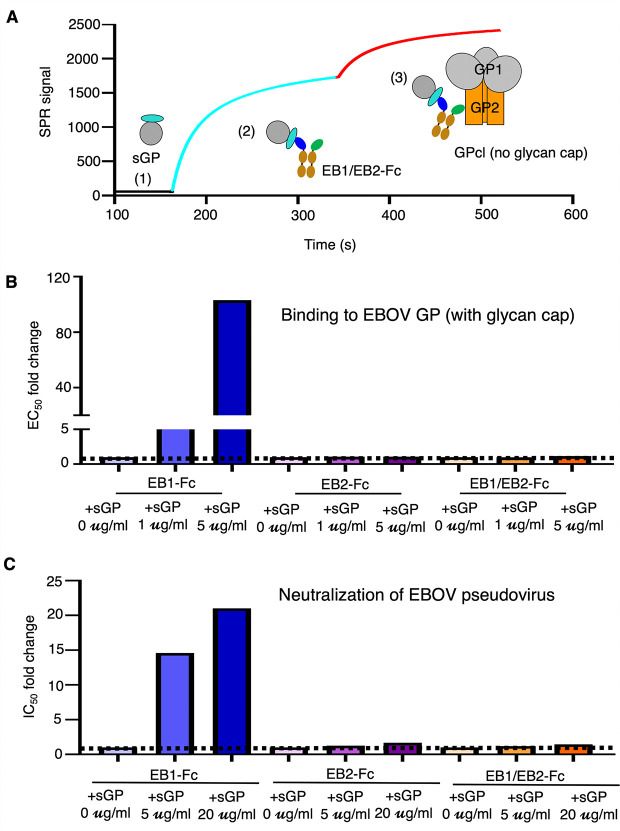
Cooperative activity of the bispecific nanobody in overcoming EBOV sGP decoy interference. **(A)** Simultaneous binding of the bispecific nanobody to GPcl (glycan cap-cleaved form of GP) and sGP, as determined by surface plasmon resonance (SPR). In step 1, the SPR sensor chip was coated with sGP (which contains the EB1-binding site but lacks the EB2-binding site) (black curve). In step 2, the bispecific nanobody (cyan curve) was injected until the SPR signal reached a plateau. In step 3, GPcl (which contains the EB2 epitope but not the EB1 epitope) (red curve) was introduced. The results confirmed that the bispecific nanobody can bind both sGP and GPcl simultaneously. **(B)** Resistance of the bispecific nanobody to sGP interference in GP binding, as measured by ELISA. Each of the three nanobodies (EB1-Fc, EB2-Fc, and EB1/EB2-Fc) was incubated with GP ectodomain in the presence of sGP at the indicated concentration. Binding was quantified by determining the half-maximal effective concentration (EC₅₀), defined as the nanobody concentration producing 50% of maximal signal. Fold changes in EC₅₀ values were calculated to compare binding in the presence versus absence of sGP. The data showed that sGP significantly impaired the binding affinity of EB1-Fc, but had no significant effect on EB2-Fc or EB1/EB2-Fc. **(C)** Resistance of the bispecific nanobody to sGP interference during EBOV pseudovirus neutralization. Each of the three nanobodies (EB1-Fc, EB2-Fc, and EB1/EB2-Fc) was tested for neutralization of EBOV pseudovirus in the presence of sGP at the indicated concentration. Neutralizing potency was expressed as IC₅₀, defined as the concentration required to inhibit pseudovirus entry by 50%. Fold changes in IC₅₀ were calculated to compare neutralization in the presence versus absence of sGP. The data demonstrated that sGP significantly reduced the neutralizing potency of EB1-Fc but had no significant effect on EB2-Fc or EB1/EB2-Fc.

To assess whether the bispecific nanobody overcomes sGP-mediated diversion, we performed three complementary assays with EB1/EB2-Fc. First, we tested whether EB1/EB2-Fc, when bound to sGP, can still engage GPcl (the glycan cap-cleaved form of GP). Using surface plasmon resonance (SPR), we immobilized sGP on a sensor chip and sequentially injected EB1/EB2-Fc followed by GPcl. The resulting SPR sensorgrams showed that EB1/EB2-Fc can simultaneously bind sGP (which contains the EB1 epitope but not the EB2 epitope) and GPcl (which contains the EB2 epitope but not the EB1 epitope), indicating that sGP-bound EB1/EB2-Fc remains capable of engaging the EB2 epitope on GPcl (Fig 4A). Second, we evaluated whether sGP interferes with EB1/EB2-Fc binding to GP containing the glycan cap. EB1/EB2-Fc binding to GP was measured by ELISA in the presence or absence of sGP, and the effect was quantified as the fold change in EC₅₀ (EC₅₀ with sGP relative to EC₅₀ without sGP). sGP had no significant impact on EB1/EB2-Fc binding to GP, whereas it markedly reduced the binding of EB1-Fc, but not EB2-Fc, to GP (Fig 4B and S2 Fig). Lastly, we examined whether sGP affects the neutralizing potency of EB1/EB2-Fc. EBOV pseudovirus entry assays were performed in the presence or absence of sGP, and the effect was quantified as the fold change in IC₅₀ (IC₅₀ with sGP relative to IC₅₀ without sGP). sGP did not significantly impair the neutralization potency of EB1/EB2-Fc (Fig 4C and S3 Fig), whereas it substantially diminished the neutralizing potency of EB1-Fc, but not EB2-Fc. Together, these results show that the bispecific nanobody engages both the EB1 and EB2 epitopes on GP, resists sGP-mediated diversion, and maintains its neutralizing activity in the presence of the sGP decoy. Thus, EB1/EB2-Fc achieves cooperative inhibition through a second mechanism: overcoming sGP-mediated immune evasion.

### In vivo characterization of anti-EBOV bispecific nanobody

**Fig 5:**
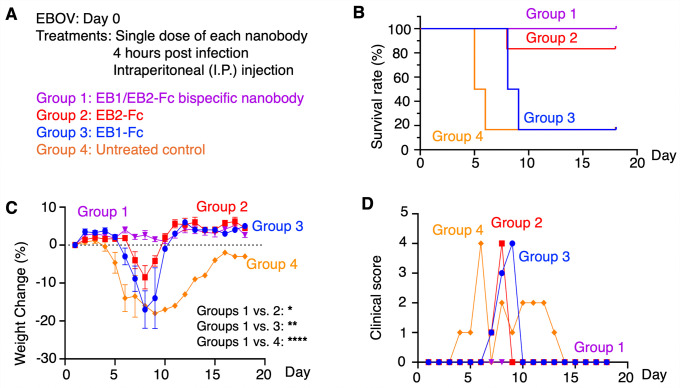
In vivo evaluation of the anti-EBOV efficacy of the bispecific nanobody in early-stage infection. **(A)** Interferon-α/β receptor–knockout mice were randomly assigned to four groups (n = 10 per group on Day 0). All mice were challenged with 100 PFU of authentic EBOV. Four hours post-infection, each group received a single 50 mg/kg dose of the indicated nanobody via intraperitoneal (I.P.) injection; PBS served as the negative control. On Day 4, four animals from each group were used for serum collection and then removed from the study, and the remaining six animals per group were monitored through Day 18. For these six animals, survival rates **(B)**, body weight changes (recorded only for surviving mice) **(C)**, and clinical scores (recorded only for surviving mice) **(D)** were monitored over 18 days. In **(C)**, data are presented as mean ± SEM (n values vary depending on the number of surviving mice on each day). P-values for weight change were calculated between the indicated experimental groups using a mixed-effects model with Geisser-Greenhouse correction, based on data collected throughout the entire experiment. ****P < 0.0001; **P < 0.01; *P < 0.05. Please note that data for individual nanobodies and untreated controls have been previously published [40] and are included here for comparison with EB1/EB2-Fc; however, data for all groups were collected concurrently.

We evaluated the in vivo efficacy of the bispecific nanobody against authentic EBOV using a stringent mouse model. In this model, wild-type EBOV was used to infect interferon-α/β-receptor-knockout mice, which are immunocompromised and therefore develop severe symptoms and experience high mortality upon infection. A single dose of EB1/EB2-Fc (50 mg/kg), which has been shown to be well tolerated owing to the high safety profile of nanobodies [40], was administered intraperitoneally (I.P.) 4 hours post-infection. For comparison, the FDA-approved antibody cocktail Inmazeb, comprising three monoclonal antibodies, is administered at a recommended dose of 50 mg/kg per antibody (150 mg/kg total) to maximize therapeutic benefit in patients, reflecting the high case fatality rate of EBOV infection [48]. In our previous study using the same treatment conditions, EB2-Fc increased survival from 17% in the untreated control group to 83% and significantly reduced weight loss and clinical scores, whereas EB1-Fc alone delayed symptom onset but did not improve survival [40] (Fig 5). In the current study, EB1/EB2-Fc provided complete protection, achieving 100% survival with no weight loss or clinical symptoms (Fig 5). These results demonstrate that the bispecific nanobody confers superior in vivo anti-EBOV efficacy compared with the individual nanobodies.

We also measured serum virus titers on Day 4. In our previous study, both EB1-Fc and EB2-Fc produced substantial reductions in viral titers, by approximately 3–4 logs relative to the control group [40] (S4 Fig). In the current study, EB1/EB2-Fc similarly reduced virus titers by ~3.5 logs compared with controls, but did not distinguish itself from the individual nanobodies by this measure. However, this readout has inherent limitations. Because control mice began to succumb by Day 5, Day 4 was the latest common sampling time, and this early measurement likely underestimates terminal viral loads, which typically surge immediately before death [49,50]. Moreover, these nanobodies are viral entry inhibitors that block new infection cycles, each requiring roughly 30 h per cell [51]; thus, Day 4 virus titers capture only the early phase of inhibition and are not strictly correlated with the peak viraemia that occurs later. Therefore, although both the bispecific nanobody and the individual nanobodies dramatically reduce serum virus titers, survival and clinical signs are more informative metrics for distinguishing anti-EBOV potency among the nanobodies in this lethal infection mouse model.

**Fig 6:**
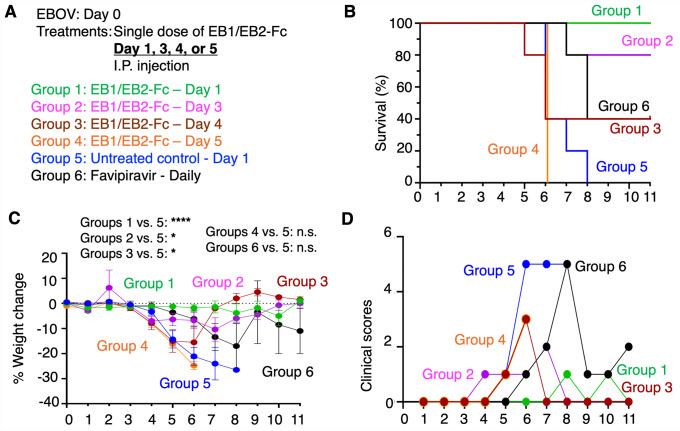
In vivo evaluation of the anti-EBOV efficacy of the bispecific nanobody in late-stage infection. **(A)** Interferon-α/β-receptor-knockout mice were divided into six groups (n = 5 per group on Day 0). All mice were challenged with 100 PFU of authentic EBOV. Mice in different groups received a single 50 mg/kg dose of EB1/EB2-Fc via **I.**P. injection, administered at the indicated time points post-infection. PBS was used as a negative control. Favipiravir, administered daily at a dose of 100 mg/kg, was included for comparison. For each group, survival rates **(B)**, body weight changes (recorded only for surviving mice) **(C)**, and clinical scores (recorded only for surviving mice) **(D)** were monitored over 11 days. In **(C)**, data are presented as mean ± SEM (n values vary depending on the number of surviving mice on each day). P-values for weight change were calculated between the indicated experimental groups using a mixed-effects model with Geisser-Greenhouse correction, based on data from the first eight days (except for groups 4 vs. 5, where only data from the first six days were used). ****P < 0.0001; *P < 0.05; n.s.: not statistically significant.

We next assessed the therapeutic potential of the bispecific nanobody in late-stage EBOV infection. A single 50 mg/kg dose of EB1/EB2-Fc was administered on Day 1, 3, 4, or 5 post-infection. In the untreated group, all mice succumbed before showing high weight loss and clinical scores (Fig 6). EB1/EB2-Fc treatment on Day 1 provided near-complete protection, resulting in 100% survival and minimal weight loss and clinical symptoms. Treatment on Day 3 offered strong protection, with 80% survival and mild weight loss and symptoms. A Day 4 dose provided significant protection (40% survival), while treatment on Day 5 offered no benefit. For comparison, favipiravir, a small-molecule EBOV inhibitor [52], was administered daily starting on Day 0. Despite the ongoing dosing, its protective effect matched that of a single EB1/EB2-Fc dose given on Day 4 in terms of survival, but with greater weight loss and more severe clinical scores (Fig 6). Overall, the bispecific nanobody showed substantial efficacy even when administered at later stages of infection.

**Fig 7:**
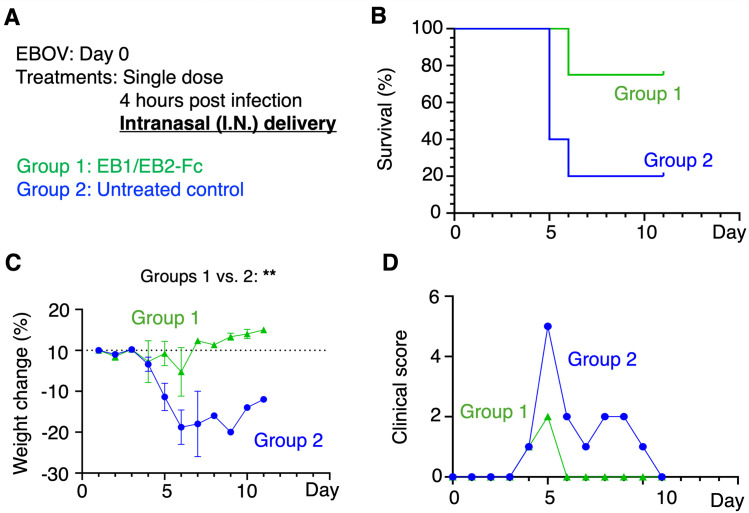
In vivo evaluation of the anti-EBOV efficacy of the bispecific nanobody administered via the intranasal route. **(A)** Interferon-α/β-receptor-knockout mice were divided into two groups (n = 5 per group on Day 0). All mice were challenged with 100 PFU of authentic EBOV. Mice in the treatment group received a single 50 mg/kg dose of EB1/EB2-Fc administered intranasally (I.N.) four hours post-infection. PBS was used as a negative control. For each group, survival rates **(B)**, body weight changes (recorded only for surviving mice) **(C)**, and clinical scores (recorded only for surviving mice) **(D)** were monitored for 11 days. In **(C)**, data are presented as mean ± SEM (n values vary depending on the number of surviving mice on each day). P-values for weight change were calculated between the indicated experimental groups using a mixed-effects model with Geisser-Greenhouse correction, based on data collected throughout the entire experiment. **P < 0.01.

Finally, we tested the in vivo efficacy of the bispecific nanobody delivered intranasally. A single 50 mg/kg dose of EB1/EB2-Fc was administered via the intranasal (I.N.) route four hours post-infection. This treatment increased survival from 20% in the untreated group to 80%, while also minimizing weight loss and clinical scores (Fig 7). These findings confirm that the bispecific nanobody retains strong in vivo anti-EBOV activity even when administered intranasally. They further suggest that, although an I.P. dose of 50 mg/kg of the bispecific nanobody was used in the mouse challenge experiments in accordance with FDA guidance, a substantially lower systemic exposure (corresponding to approximately 5.4% of the 50 mg/kg I.P. dose achieved via the I.N. route; see below for the bioavailability of I.N. compared with I.P. delivery) is sufficient to confer robust protection in EBOV-challenged mice.

### Pharmacokinetics and in vitro stability of anti-EBOV bispecific nanobody

**Fig 8:**
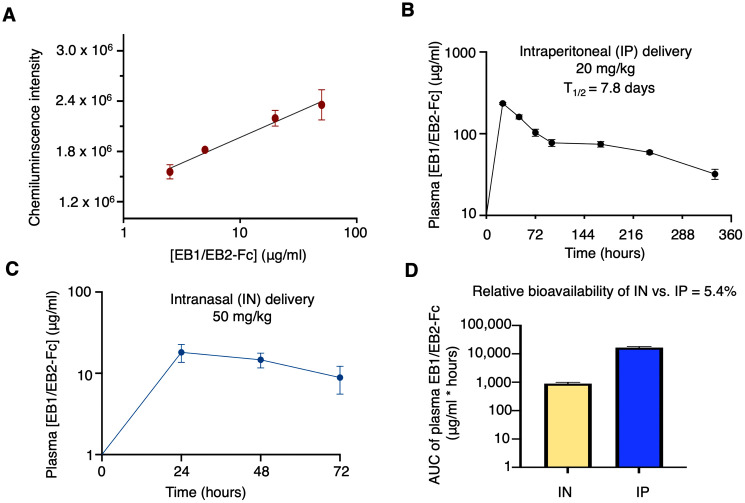
Pharmacokinetics of the bispecific nanobody in mice. Human neonatal Fc receptor (hFcRn) transgenic mice were used in this study. **(A)** A log-linear relationship between EB1/EB2-Fc concentrations and chemiluminescence intensities was established by ELISA and used in calculating the plasma concentrations of EB1/EB2-Fc. **(B)** EB1/EB2-Fc was administered through the **I.**P. route to mice (n = 3), its plasma concentrations at different time points were recorded, and its plasma half-life was calculated. **(C)** EB1/EB2-Fc was administered through the **I.**N. route to mice (n = 3) and its plasma concentrations at different time points were recorded. **(D)** The relative bioavailability of **I.**N. delivery compared to **I.**P. injection was determined using the ratio of the total exposure to EB1/EB2-Fc, represented by the area under the curve (AUC) values. Data are presented as mean ± SEM (n = 3).

To evaluate the pharmacokinetics (PK) of the bispecific nanobody, we measured its half-life in mice. First, we established a log-linear relationship between EB1/EB2-Fc concentrations and chemiluminescence intensities using ELISA (Fig 8A). A single dose of EB1/EB2-Fc was then I.P. administered at 20 mg/kg. A key factor influencing the half-life of Fc-domain-fused proteins is the binding affinity between the Fc domain and the neonatal Fc receptor (FcRn) in animal models [53]. While human Fc binds well to murine FcRn [54], it is most compatible with its native receptor, human FcRn (hFcRn). Therefore, hFcRn transgenic mice were used in this study. In these mice, plasma EB1/EB2-Fc levels peaked at 234.6 µg/ml 24 hours post-injection and gradually declined to 32.2 µg/ml over the following 14 days (Fig 8B). Based on the PK profile, the estimated plasma half-life of EB1/EB2-Fc was 7.8 days.

To evaluate the bioavailability of intranasal (I.N.) delivery, a single 50 mg/kg dose of EB1/EB2-Fc was administered to hFcRn transgenic mice via the I.N. route. Plasma EB1/EB2-Fc levels peaked at 18.1 µg/ml 24 hours post-injection and gradually declined to 8.9 µg/ml over the following 3 days (Fig 8C). The total exposure to the bispecific nanobody, represented by the area under the curve (AUC), was calculated for both I.P. and I.N. administrations. Based on these AUC values, the relative bioavailability of I.N. delivery compared to I.P. was determined to be 5.4% (Fig 8D).

**Fig 9:**
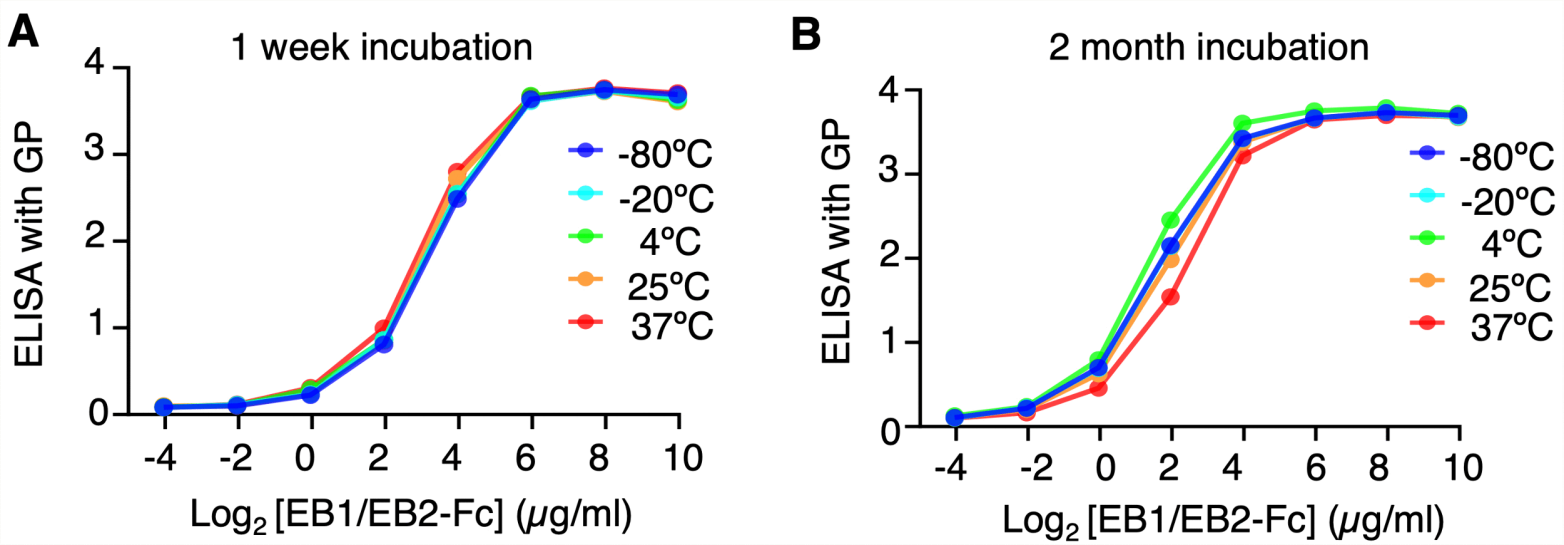
In Vitro Stability of the bispecific nanobody. ELISA was performed to evaluate the effect of storage conditions on the binding affinity of EB1/EB2-Fc to recombinant EBOV GP ectodomain. Data are presented as mean ± SEM (n = 3).

To assess in vitro stability, EB1/EB2-Fc was incubated for either one week or two months at -80°C, 4°C, 25°C, or 37°C, and its binding to EBOV GP was evaluated by ELISA. Using -80°C storage as the reference, EB1/EB2-Fc retained full binding activity after one week and nearly full activity after two months at 37°C (Fig 9). These findings demonstrate that EB1/EB2-Fc possesses excellent in vitro stability.

## Discussion

EBOV remains a major threat to global health and national security due to its high fatality rates and recurring outbreaks. Although two antibody therapies and one vaccine have been approved, these interventions offer only modest reductions in mortality and are hindered by their reliance on injectable administration, high production costs, and cold-chain logistics [7–9]. These limitations are particularly problematic in regions where EBOV outbreaks occur, which have warm climates and limited access to healthcare facilities or refrigeration [10]. To overcome these challenges, we aimed to develop novel anti-EBOV therapeutics with both high potency and broad accessibility.

We engineered a bispecific nanobody, Nanosota-EB1/EB2-Fc, by fusing two individual nanobodies, Nanosota-EB1 and Nanosota-EB2, to a human Fc domain. This construct was designed to combine and maximize the antiviral activities of EB1 and EB2, which respectively target glycan cap proteolysis and membrane fusion functions of EBOV GP. EB1 also carries strong ADCC potential because its binding epitope on GP is highly exposed. Structural and biochemical analyses demonstrated that the bispecific nanobody cooperatively inhibits GP function by more effectively blocking glycan cap cleavage and successfully evading the sGP decoy mechanism. Cooperative binding of EB1 and EB2 to GP stabilizes the glycan cap and prevents its proteolytic removal, thereby limiting exposure of the RBS. As a result, although the bispecific nanobody combines a strong neutralizer (EB2) with a weak neutralizer (EB1) and might be expected to exhibit intermediate activity, it instead displays neutralizing potency matching that of EB2-Fc (containing two copies of EB2) alone in vitro. Meanwhile, the bispecific nanobody resists the sGP decoy by retaining its ability to bind GP in the presence of sGP, overcoming viral immune evasion and allowing EB1 to exert its anti-EBOV activity. Overall, the bispecific nanobody preserves the strong neutralizing activity of EB2 and the proteolysis interference activity of EB1, both of which are manifested in vitro and in vivo, and it also retains the strong ADCC potential of EB1, which is manifested only in vivo.

Due to its novel mechanisms of action, the bispecific nanobody shows strong promise as a highly potent therapeutic candidate against EBOV. In our stringent preclinical model using immunocompromised mice, EBOV infection resulted in 80–100% mortality, with fatalities typically beginning around Day 5 post-infection. A single dose of the bispecific nanobody I.P. administered at early stages (4 hours or Day 1 post-infection) provided complete or near-complete protection, achieving 100% survival with minimal weight loss and clinical symptoms. At Day 3, a single dose still offered strong protection with 80% survival and small weight loss and clinical symptoms. Even at Day 4, one day before mice succumb, the nanobody provided significant protection, with 40% survival and reduced weight loss and clinical symptoms. Although we were unable to obtain FDA-approved antibody drugs for direct comparison, we tested Favipiravir, a known EBOV polymerase inhibitor [52], as a benchmark. When administered daily, Favipiravir produced the same survival rate as a single Day 4 dose of the bispecific nanobody but resulted in worse weight loss and clinical symptoms, highlighting both the stringency of our preclinical model and the superior potency of the bispecific nanobody. These results demonstrate that the bispecific nanobody offers strong protection against both early and late EBOV infections in a stringent preclinical model, though further validation in other models, particularly non-human primates, is needed.

Despite having nearly identical neutralizing potency to EB2-Fc in vitro, the bispecific nanobody outperforms EB2-Fc in vivo. Because its in vitro potency already captures the cooperativity between EB1 and EB2, the superior in vivo activity of the bispecific nanobody over EB2-Fc likely arises from additional antiviral effects, such as ADCC, that are present in the bispecific nanobody but absent in EB2-Fc. By combining an exposed epitope targeting arm (EB1) with a human IgG Fc domain, the bispecific nanobody should be able to engage Fc-mediated antiviral mechanisms, including ADCC, through simultaneous binding of the exposed GP epitope and Fcγ receptors (FcγRs) on immune cells [24]. However, in the current study this Fc-mediated potential may not have been fully manifested because two factors likely dampened these effects: (i) production in human Expi293F cells, which typically yields Fc glycans associated with reduced ADCC [55], and (ii) the limited cross-species affinity of human Fc for mouse FcγRs [56]. To evaluate the full contribution of Fc-driven antiviral mechanisms, we will express the bispecific nanobody in CHO cells to generate an ADCC-enhanced Fc variant and assess its efficacy in human FcγR transgenic mice [56,57]. These future studies will determine whether Fc-driven activity can further augment the already potent anti-EBOV efficacy of the bispecific nanobody reported here, thereby strengthening its potential as a next-generation anti-EBOV therapeutic.

In addition to its antiviral potency, the bispecific nanobody shows strong promise as a widely accessible therapeutic. Existing vaccines and antibody treatments are limited by their reliance on injection and cold-chain logistics. In contrast, our nanobody was highly effective when delivered intranasally (I.N.), providing strong protection in EBOV-infected mice and enabling needle-free administration in home or field settings. The nanobody’s human IgG Fc tag increases its molecular size beyond the renal clearance threshold [32], resulting in a serum half-life of 7.8 days in mice - suitable for single-dose therapeutic use. Despite this increase in size, the Fc-domain-fused bispecific nanobody remains about half the size of a full human IgG antibody, which substantially enhances its intranasal delivery efficiency. Pharmacokinetic analysis showed that I.N. delivery achieved 5.4% bioavailability relative to I.P. injection, with plasma concentrations reaching 18.1 µg/ml at 24 hours. The combination of an 18.1 µg/ml plasma concentration at 24 hours after I.N. delivery and a 7.8-day half-life ensures that plasma levels remain well above the 29 ng/ml IC₅₀ of the bispecific nanobody for an extended period, accounting for its strong in vivo efficacy via the I.N. route. Moreover, the nanobody demonstrated exceptional in vitro stability. After incubation at 37°C for two months, it retained nearly all of its target-binding activity, indicating that transport and storage without cold-chain infrastructure is feasible. This in vitro stability likely stems from its heavy-chain-only structure, which avoids the denaturation issues commonly seen in conventional antibodies with separate heavy and light chains. Taken together, the bispecific nanobody’s needle-free delivery and cold-chain independence make it far more accessible than current vaccines and antibody therapeutics - especially in regions affected by EBOV outbreaks, where limited clinical infrastructure, warm weather, and lack of refrigeration present major logistical barriers.

Although the bispecific nanobody exhibits broad-spectrum anti-EBOV activity by potently neutralizing four major EBOV strains, nanobody resistance mutations may emerge during treatment and compromise its efficacy. A key advantage of bispecific nanobodies is that they engage two distinct epitopes on the same GP, so the virus would need to acquire simultaneous mutations at both sites to escape neutralization, an event that is far less likely than resistance to a monospecific nanobody [58–60]. To further limit the risk of viral escape, we recently developed a structure-guided in vitro evolution strategy that enables rapid adaptation of nanobodies to new viral variants [37]. This approach introduces targeted mutations at key nanobody residues located near viral mutation sites and uses phage display to comprehensively sample all possible mutation combinations at these positions, allowing the entire adaptation cycle to be completed in under two weeks. This strategy highlights another advantage of nanobodies over conventional human antibodies: their single-domain architecture simplifies phage display, permits efficient bacterial expression, and streamlines functional screening. Together, the inherent advantage of bispecific nanobody design and our novel nanobody engineering approach provide powerful tools to limit and overcome potential nanobody-resistant mutations in EBOV GP.

Like antibody cocktails, nanobody cocktails have several advantages over single nanobodies as potential anti-EBOV therapeutics, including cooperative inhibition of EBOV entry and a reduced risk of viral escape. Our previous study showed that a cocktail of EB1-Fc and EB2-Fc (25 mg/kg each) provided similar protection to EB2-Fc alone (50 mg/kg) in EBOV-challenged mice [40], indicating that cooperativity in neutralizing EBOV entry is present but modest and weaker than that achieved by the bispecific nanobody. This limited cooperativity likely stems from cooperative inhibition of glycan cap proteolysis by the two nanobodies. However, because of the sGP decoy effect, not all EB1-Fc molecules successfully bind GP on viral particles, which restricts their ability to block glycan cap proteolysis and to mediate ADCC. This explains why the bispecific nanobody provides complete protection in EBOV-challenged mice during early-stage infection, whereas the EB1-Fc/EB2-Fc cocktail, like EB2-Fc alone, confers strong but incomplete protection. Moreover, because the bispecific nanobody is a single protein molecule, manufacturing, storage, transport, and product quality control are substantially easier than for cocktails. Overall, the bispecific nanobody offers several advantages over nanobody cocktails as a potential anti-EBOV therapeutic.

In summary, we developed a bispecific nanobody that targets two distinct epitopes on EBOV GP, enabling cooperative inhibition of viral entry, immune evasion, and Fc-mediated antiviral effector mechanisms. The nanobody demonstrated strong protective efficacy in a stringent EBOV mouse model, remained effective even when administered at late stages of infection or via the intranasal route, and exhibited excellent in vitro stability. With its potent antiviral activity, needle-free delivery capability, and cold-chain independence, this novel bispecific nanobody represents a highly promising next-generation therapeutic candidate for future EBOV outbreak preparedness and response.

## Materials and methods

### Ethics statement

This study was performed in strict accordance with the recommendations in the Guide for the Care and Use of Laboratory Animals of the National Institutes of Health. All of the animals were handled according to approved institutional animal care and use committee (IACUC) protocols of the Boston University (protocol number: 201900062) and the University of Tennessee Health Science Center (IACUC protocol no. 22–0371).

### Cell lines, plasmids, and virus

HEK293T and Huh7 cells (American Type Culture Collection, ATCC) were cultured in Dulbecco’s Modified Eagle Medium (DMEM) supplemented with 10% fetal bovine serum, 2 mM L-glutamine, 100 U/ml penicillin, and 100 µg/ml streptomycin. Expi293F cells (ThermoFisher) used for protein expression were maintained in Expi293 Expression Medium (ThermoFisher). No commonly misidentified cell lines were used in this study.

The EBOV GP genes (Mayinga, NCBI Reference Sequence NC_002549.1; Makona, GenBank: AIE11800.1; Ituri, GenBank: AYN74184.1; Mbandaka, GenBank: WWV92555.1) were synthesized (GenScript). For pseudovirus packaging, the full-length EBOV GP gene was cloned into the pcDNA3.1(+) vector with a C-terminal C9 tag. For protein expression, the gene encoding the EBOV GP ectodomain lacking the mucin-like domain (residues 313–463 deleted) (referred to as the GP ectodomain for simplicity) was cloned into the Lenti-CMV vector (Vigene Biosciences) with a C-terminal foldon trimerization motif followed by a His tag. The gene encoding EBOV sGP (residues 1–305) was similarly cloned into the Lenti-CMV vector with a C-terminal His tag. Plasmids encoding Fc-domain-fused nanobodies were cloned into the Lenti-CMV vector with an N-terminal tPA signal peptide and a C-terminal human Fc domain. To generate the bispecific nanobody Nanosota-EB1/EB2-Fc, a “knobs-into-holes” strategy was employed, introducing the T366Y and Y407T mutations into the Fc regions of the Lenti-CMV-Nanosota-EB1 and Lenti-CMV-Nanosota-EB2 constructs, respectively [41].

Authentic EBOV (strain H.sapiens-tc/COD/1976/Yambuku-Mayinga) was used to infect Huh7 cells for in vitro assays and interferon-α/β-receptor-knockout mice for in vivo assays. All experiments involving infectious EBOV were conducted in approved Biosafety Level 4 laboratories at the National Institute of Allergy and Infectious Diseases (for in vitro assays) and the National Emerging Infectious Disease Laboratories at Boston University (for in vivo assays).

### Protein expression and purification

The EBOV GP ectodomain, EBOV sGP, and Fc-domain-fused nanobodies were expressed and purified from mammalian cells as previously described [34,61]. Briefly, plasmids encoding each of these proteins were transiently transfected into Expi293F cells using polyethylenimine (PEI; Polysciences). For expression of Nanosota-EB1/EB2-Fc, equal amounts of expression plasmids for EB1-Fc (carrying the T366Y mutation in the Fc region) and EB2-Fc (carrying the Y407T mutation in the Fc region) were co-transfected into Expi293F cells. Three days post-transfection, all proteins were harvested from the culture supernatants. The EBOV GP ectodomain and sGP were purified using a Ni-NTA affinity column followed by further purification on a Superose 6 increase 10/300 gel filtration column (Cytiva). Fc-domain-fused nanobodies were purified using a Protein A column followed by further purification on a Superdex 200 increase 10/300 gel filtration column (Cytiva). To generate EBOV GPcl (glycan cap-cleaved form of GP ectodomain), 3 mg of EBOV GP ectodomain was digested with 15 µg of thermolysin L (Sigma-Aldrich) overnight at room temperature and then purified on a Superose 6 increase 10/300 column (Cytiva).

To prepare the ternary complex of EBOV GP ectodomain, EB1, and EB2, the GP ectodomain was incubated with an excess of His-tagged EB1 and EB2 at room temperature for 1 hour. The complex was then purified using a Superose 6 increase 10/300 column (Cytiva).

### Neutralizing potency of the bispecific nanobody against authentic EBOV

The neutralizing potency of EB1/EB2-Fc against authentic EBOV was assessed as previously described [40]. EB1/EB2-Fc was diluted to specified concentrations in cell culture medium and incubated with the virus for 60 minutes. Infection levels were evaluated using an immunofluorescence assay. Cell nuclei were stained with Hoechst dye (ThermoFisher), and nuclei counts were used as a proxy for total cell counts. The efficacy of EB1/EB2-Fc was determined by calculating the concentration required to reduce the number of infected cells by 50% (IC₅₀) relative to the virus-only control group.

### Neutralizing potency of the bispecific nanobody against EBOV pseudoviruses

The EBOV pseudovirus entry assay was performed as previously described [40]. Briefly, EBOV pseudoviruses were generated by co-transfecting HEK293T cells with a pcDNA3.1(+) plasmid encoding full-length EBOV GP, the helper plasmid psPAX2, and the reporter plasmid plenti-CMV-luc. After 72 hours, pseudoviruses were harvested and incubated with each nanobody at various concentrations for 1 hour at 37°C, then used to infect Huh7 cells. Following a 48-hour incubation, the cells were lysed, and aliquots of the lysates were transferred to new plates. A luciferase substrate was added, and Relative Light Units (RLUs) were measured using an EnSpire plate reader (PerkinElmer). Nanobody potency was assessed by determining the concentration required to inhibit pseudovirus entry by 50% (IC₅₀). To evaluate the effect of sGP on nanobody neutralization, the assay was repeated as described above, with recombinant sGP added at two concentrations (5 µg/ml and 20 µg/ml).

### Cryo-EM data collection, data processing, model building and refinement

4 µL of the purified ternary complex of EBOV GP ectodomain, EB1, and EB2 (~1.25 µM) was applied to freshly glow-discharged Quantifoil R1.2/1.3 300-mesh copper grids (EM Sciences). Grids were blotted for 4 seconds at 22°C under 100% chamber humidity and plunge-frozen in liquid ethane using a Vitrobot Mark IV (FEI). Cryo-EM data were collected using a Latitude-S system (Gatan) equipped with a K3 direct electron detector and a Biocontinuum energy filter (Gatan). Movies were recorded at a nominal magnification of 130,000 × , corresponding to a pixel size of 0.664 Å. Data collection statistics are summarized in S1 Table.

Cryo-EM data were processed using cryoSPARC v3.3.2 [62], with the workflow summarized in S1 Fig. Briefly, dose-fractionated movies were aligned using Patch motion correction with MotionCor2 [63] and CTF parameters were estimated using Patch CTF estimation with CTFFIND-4.1.13 [64]. Particles were picked using the Blob picker in cryoSPARC v3.3.2. Junk particles were eliminated through three rounds of 2D classification. Particles from well-defined 2D classes were used for ab initio reconstruction into three maps. These initial models served as starting references for heterogeneous refinement (3D classification). The best 3D classes were then refined using homogeneous, non-uniform, and CTF refinement procedures to generate the final maps, with C3 symmetry applied for the ternary complex. Map resolutions were determined using gold-standard Fourier shell correlation (FSC) at the 0.143 threshold between the two half-maps.

Initial model building of the ternary complex was performed in Coot v0.8.9 [65], using the GP ectodomain from PDB entry 9BSU as the starting model. The initial nanobody models were taken from PDB entries 9BSU and 9BSV, respectively, and fitted into the density map. Multiple rounds of refinement in Phenix v1.16 [66] and manual adjustments in Coot were carried out to generate the final model. Model and map statistics are summarized in S1 Table. All structural figures were prepared using UCSF ChimeraX v0.93 [67].

### Glycan cap cleavage

Proteolysis of the GP glycan cap was performed as previously described [40]. Briefly, 60 µg of EBOV GP ectodomain complexed with EB1/EB2-Fc was treated with 0.25 µg of thermolysin L (Sigma-Aldrich) at 37°C for varying durations (5, 15, 30, or 60 minutes). As a control, 60 µg of EBOV GP ectodomain alone was treated under the same conditions. At each time point, aliquots were immediately mixed with SDS-PAGE loading buffer and boiled for 10 minutes to terminate the reaction. All samples were then analyzed by non-reducing SDS-PAGE.

### Surface plasmon resonance

Surface plasmon resonance (SPR) was performed to assess the simultaneous binding of EB1/EB2-Fc to sGP and GPcl. Recombinant sGP was immobilized on a CM5 sensor chip (Cytiva) via chemical crosslinking. EB1/EB2-Fc (40 µg/ml) was first injected, followed by injection of GPcl at the same concentration. SPR signals were recorded to monitor binding.

### ELISA to evaluate the effect of sGP on the bispecific nanobody binding to EBOV GP

An ELISA was performed to assess the effect of recombinant sGP on the binding of three nanobodies—EB1-Fc, EB2-Fc, and the bispecific EB1/EB2-Fc—to recombinant EBOV GP ectodomain. ELISA plates were coated with GP ectodomain, and serial dilutions of each nanobody (starting at 10 µg/ml with 4-fold dilution steps) were added. Recombinant sGP was then introduced at two concentrations (1 µg/ml and 5 µg/ml). After incubation, a horseradish peroxidase (HRP)-conjugated anti-Fc antibody (1:3,000; Sigma-Aldrich) was added. The ELISA substrate (Invitrogen) was then applied, and the reactions were stopped using 1N H₂SO₄. Absorbance at 450 nm (A₄₅₀) was measured using a Synergy LX Multi-Mode Reader (BioTek). Binding interactions were quantified by calculating the half-maximal effective concentration (EC₅₀), defined as the concentration of nanobody required to elicit 50% of the maximum signal.

### Anti-EBOV efficacy of the bispecific nanobody in mice

The in vivo efficacy of EB1/EB2-Fc against EBOV was evaluated in an interferon-α/β-receptor-knockout mouse model across three experiments.

In the first experiment, 40 mice were randomized into four groups (n = 10). Authentic EBOV and nanobodies were administered via I.P. injection. All mice were challenged with 100 PFU of EBOV in 100 µL PBS buffer on Day 0. Four hours post-infection, mice received one of the indicated nanobodies at a dose of 50 mg/kg. Survival, body weight, and clinical scores were monitored for 18 days. On Day 4, four animals per group (2 males and 2 females) were randomly selected, euthanized, and serum was collected; these animals were excluded from the survival, body weight, and clinical score analyses. The remaining animals continued in the study under the same conditions until Day 18, when the experiment was terminated. Data for the individual nanobodies and the untreated control group have been previously published [40] and are referenced here for comparison to EB1/EB2-Fc. However, all groups were tested in parallel.

To measure serum viral loads, sera were inactivated using Trizol LS (Invitrogen) and used to determine genome copy number (GN). Total RNA was isolated with the Direct-zol RNA Miniprep Kit (Zymo Research) according to the manufacturer’s instructions. RNA concentration and quality were assessed using a NanoDrop spectrophotometer (NanoDrop Technologies). EBOV-specific primers (IDT, 5′-CATGCGTACCAGGGAGATTAC-3′, 5′-ACTCCATCACGCTTCTTGAC-3′) and an EBOV-specific probe (IDT, 5′-/56-FAM/TCAAGTATT/ZEN/TGGAAGGGCACGGGT/3IABkFQ/-3′) were used for reverse-transcription quantitative PCR (RT-qPCR) with the Luna One-Step Universal Probe RT-qPCR Kit (New England Biolabs), following the manufacturer’s recommendations, on a Bio-Rad instrument using Bio-Rad Maestro software for analysis. A standard curve was generated from a synthetic EBOV RNA standard using 10-fold serial dilutions in water. Each sample, including standards, was run in duplicate along with a non-targeting control. Duplicate values were averaged, and the standard curve was used to back-calculate GN for the serum samples.

In the second experiment, 30 mice were randomized into 6 groups (n = 5). EBOV, EB1/EB2-Fc, and favipiravir were administered via I.P. injection. All mice received 100 PFU of EBOV in 100 µL PBS buffer on Day 0. EB1/EB2-Fc was administered at a dose of 50 mg/kg at the following time points post-infection: 4 hours (group 1), Day 1 (group 2), Day 3 (group 3), or Day 4 (group 4). Group 5 (untreated control) received PBS at 4 hours post-infection, and group 6 received favipiravir at 100 mg/kg daily, starting on Day 0. Survival, body weight, and clinical scores were monitored for 11 days.

In the third experiment, 10 mice were randomized into 2 groups (n = 5). EBOV was administered via I.P. injection, while EB1/EB2-Fc was delivered intranasally (I.N.). All mice were infected with 100 PFU of EBOV in 100 µL PBS buffer on Day 0. Four hours post-infection, mice in the treatment group received 50 mg/kg of EB1/EB2-Fc via the I.N. route, while control mice received PBS. Survival, body weight, and clinical scores were monitored for 11 days.

### Statistical analysis of mouse study

GraphPad Prism (version 10.3.0) was used for all data analyses and statistical evaluations. Survival curves were generated using the Kaplan-Meier method. Percent weight change for each animal was calculated relative to its baseline weight. P-values for weight change were calculated between the indicated experimental groups using a mixed-effects model with Geisser-Greenhouse correction. Clinical scores were recorded as the highest score observed for each group on each day. Viral loads were quantified and expressed as genome numbers per milliliter (GN/ml). GN/ml values were log-transformed, and P values for viral loads were calculated by comparing the control group with each treatment group using an unpaired, two-tailed Student’s t test.

### Pharmacokinetics of the bispecific nanobody in mice

Human FcRn transgenic mice (Jackson Laboratory) received EB1/EB2-Fc either via I.P. at a dose of 20 mg/kg or via the intranasal (I.N.) route at 50 mg/kg. At 24–336 hours post-administration, small blood samples were collected from the tail vein and plasma was stored at -80°C until analysis.

A calibration curve for quantifying EB1/EB2-Fc in plasma was established, demonstrating a log-linear relationship between plasma nanobody concentrations and chemiluminescence intensities, which was used for subsequent ELISA measurements. To minimize interference from endogenous plasma proteins, plasma samples were diluted at least 100-fold in PBS prior to ELISA.

ELISA was performed to measure EB1/EB2-Fc concentrations in mouse plasma. Plates were coated with donkey anti-human IgG Fc antibody (Jackson ImmunoResearch) at 4°C overnight. After washing with PBS, plates were blocked with 2% BSA at room temperature for 1 hour. Following PBST (PBS + 0.05% Tween-20) washes, mouse plasma samples were added and incubated at room temperature for 1 hour. Plates were then washed again with PBST and incubated with HRP-conjugated goat anti-human IgG Fc antibody (1:20,000 dilution; Jackson ImmunoResearch) for 1 hour at room temperature. After sequential washes with PBST and PBS, SuperSignal Femto substrate (ThermoFisher) was added for 1 minute, and chemiluminescence was measured using a BioTek Synergy H1 plate reader (Agilent).

### In vitro stability of the bispecific nanobody

EB1/EB2-Fc was incubated at various temperatures for either 1 week or 2 months. ELISA was then performed to assess its residual target-binding activity, as previously described [40]. Briefly, ELISA plates were coated with recombinant EBOV GP ectodomain, followed by the addition of serially diluted EB1/EB2-Fc. Subsequent steps were carried out as described in the ELISA procedure above for evaluating the effect of sGP on bispecific nanobody binding to EBOV GP.

## Disclaimer note

The content of this publication does not necessarily reflect the views or policies of the US Department of Health and Human Services (DHHS) or of the institutions and companies affiliated with the authors.

## Supporting information

S1 Fig Cryo-EM image processing and map reconstruction workflow for the ternary complex of EBOV GP ectodomain, Nanosota-EB1, and Nanosota-EB2. Representative raw cryo-EM images and 2D class averages of the complex are shown. 3D refinement using particles from high-quality 3D classes with C1 symmetry yielded a 3.15-Å map, revealing three copies each of EB1 and EB2. Further 3D refinement with C3 symmetry increased the map resolution to 2.92-Å and improved the densities for the nanobodies. The final maps, half-map FSC curves, angular distribution plot, and corresponding local-resolution maps are shown within the dashed black box.
(TIF)

S2 Fig Impacts of sGP on the target-binding affinities of Nanosota-EB1-Fc, Nanosota-EB2-Fc, and Nanosota-EB1/EB2-Fc. See the legend for Fig 4B for details. Data are presented as mean ± SEM (n = 3).
(TIF)

S3 Fig Impacts of sGP on the neutralizing potencies of Nanosota-EB1-Fc, Nanosota-EB2-Fc, and Nanosota-EB1/EB2-Fc. See the legend for Fig 4C for details. Data are presented as mean ± SEM (n = 3).
(TIF)

S4 Fig Viral loads in EBOV-challenged mice measured on Day 4 post infection. Comparisons between the control group and each treatment group were performed using an unpaired, two-tailed Student’s t test. Error bars represent SEM (n = 4). ****p < 0.0001. Please note that data for individual nanobodies and untreated controls have been previously published [40] and are included here for comparison with EB1/EB2-Fc; however, data for all groups were collected concurrently.
(TIF)

S1 Table Cryo-EM data collection, model refinement and validation statistics. (DOCX)
