# Supplementary material for: A highly potent and broadly accessible bispecific nanobody for the treatment of ebola virus infections
Source: PLoS Pathog. 2026 Jan 27;22(1):e1013878. doi: 10.1371/journal.ppat.1013878 (PMC12858065; doi:10.1371/journal.ppat.1013878)
Supplement: S1 Table — (DOCX) [file ppat.1013878.s005.docx]

**S1 Table. Cryo-EM data collection, model refinement and validation statistics.**

|  | EBOV GP/EB1/EB2 ternary complex |
| --- | --- |
| **Data collection and processing** |  |
| Magnification | 130,000 |
| Voltage (kV) | 300 |
| Electron exposure (e–/Å^2^) | 50.00 |
| Defocus range (μm) | -1.0 ~ -2.0 |
| Pixel size (Å) | 0.664 |
| Symmetry imposed | C3 |
| Initial particle images (no.) | 3,765,265 |
| Final particle images (no.) | 62,575 |
| Map resolution (Å)  FSC threshold (half-maps) | 2.92  0.143 |
|  |  |
| Map resolution range (Å) | 2.6–7.2 |
|  |  |
| **Model refinement** |  |
| Initial model used (PDB code) | 9BSU/9BSV |
| Model resolution (Å)  FSC threshold (model-map) | 3.1  0.5 |
|  |  |
| Model resolution range (Å) | 43.3-2.8 |
| Map sharpening *B* factor (Å^2^) | -83.5 |
| Model composition  Non-hydrogen atoms  Protein residues  Ligands |  |
|  | 13954 |
|  | 1770 |
|  | 20 |
| *B* factors (Å^2^)  Protein  Nucleotide  Ligand |  |
|  | 59.40 |
|  |  |
|  | 69.53 |
| R.m.s. deviations  Bond lengths (Å)  Bond angles (°) |  |
|  | 0.005 |
|  | 1.031 |
| Validation  MolProbity score  Clashscore  Poor rotamers (%) | 1.58  4.27  0.21 |
|  |  |
|  |  |
|  |  |
| Ramachandran plot  Favored (%)  Allowed (%)  Disallowed (%) | 94.59  5.01  0.40 |
|  |  |
|  |  |
|  |  |
